# Comparative study of robotic-assisted vs. laparoscopic surgery for colorectal cancer: a single-center experience

**DOI:** 10.3389/fonc.2024.1507323

**Published:** 2025-01-07

**Authors:** Wenpeng Wang, Jia Liu, Jiefu Wang, Li Li, Dalu Kong, Junfeng Wang

**Affiliations:** ^1^ Department of Colorectal Oncology, Tianjin Medical University Cancer Institute and Hospital, National Clinical Research Center for Cancer, Tianjin’s Clinical Research Center for Cancer, Tianjin Key Laboratory of Digestive Cancer, Tianjin, China; ^2^ NHC Key Laboratory of Hormones and Development, Tianjin Key Laboratory of Metabolic Diseases, Chu Hsien-I Memorial Hospital & Tianjin Institute of Endocrinology, Tianjin Medical University, Tianjin, China

**Keywords:** robotic surgery, laparoscopic surgery, colectomy, proctectomy, colorectal cancer

## Abstract

**Background:**

Colorectal cancer (CRC) surgeries are commonly performed using either robotic-assisted colorectal surgery (RACS) or laparoscopic colorectal surgery (LCS). This study aimed to compare clinical and surgical outcomes between RACS and LCS for CRC patients.

**Methods:**

We included 225 patients from Tianjin Medical University Cancer Institute & Hospital (TJMUCH) between January 2021 and June 2024, divided into RACS (n=82) and LCS (n=143) groups. Data on demographics, clinicopathological variables, surgical parameters, and perioperative outcomes were analyzed. Statistical significance was set at p < 0.05.

**Results:**

RACS was associated with longer surgery durations (median: 218.5 vs. 165 minutes) and greater blood loss (median: 100 vs. 50 mL) compared to LCS (p < 0.001 for both). Additionally, the median hospitalization cost was notably higher for RACS at 117,822 RMB compared to 78,174 RMB for LCS (p < 0.0001). RACS was used more frequently for proctectomy (87.80% vs. 72.48%). No significant differences were found in lymph node dissection (LND), postoperative hospital stay, conversion to open surgery, or postoperative complications between the groups (p > 0.05). Anastomotic leakage was the most common complication in both groups (RACS: 3.66%, LCS: 4.20%), with no significant difference in incidence (p = 0.876). To reduce bias due to surgical site, cases of rectal and sigmoid colon cancer were singled out. Hierarchical analysis showed significant differences still remained in surgical duration, blood loss, and surgical site distribution for proctectomy and sigmoid colon resection (p < 0.001). RACS did not show a clear advantage in surgical field exposure or tissue retraction.

**Conclusion:**

RACS, despite superior visualization, involved longer operative times and more blood loss than LCS. Both techniques had similar clinical outcomes, with LCS offering specific technical advantages.

## Introduction

Colorectal cancer (CRC) remains one of the most prevalent and lethal malignancies globally, posing a significant public health challenge despite advancements in detection and treatment strategies (1, 2). Over the past few decades, minimally invasive surgical techniques have revolutionized the management of CRC, leading to the widespread adoption of laparoscopic surgery (3). Laparoscopy offers numerous benefits, including reduced postoperative pain, decreased postoperative intra-abdominal or incision site infections, shorter hospital stays, and faster recovery (4). However, the technical challenges of laparoscopic surgery, such as limited instrument articulation, two-dimensional visualization, and the need for increased collaboration among medical staff, have prompted the development and integration of robotic-assisted surgery.

Robotic-assisted colorectal surgery (RACS) has emerged as a promising alternative to traditional laparoscopy, offering enhanced dexterity, three-dimensional visualization, and improved ergonomics (5–7). Robotic platforms, such as the da Vinci system, provide surgeons with greater precision and control, potentially overcoming the constraints of laparoscopy (8). The introduction of robotic platforms into colorectal surgery contributes to overcome the limitations of laparoscopic colorectal surgery (LCS) and potentially improve surgical outcomes (8, 9). Despite these theoretical advantages, the clinical superiority of RACS over LCS remains a topic of ongoing debate. While RACS has been associated with improved surgeon comfort and enhanced visualization, concerns have been raised regarding its longer operative times, higher costs, and potential for increased blood loss (9–11). Moreover, the evidence on whether RACS improves oncological outcomes, such as lymph node dissection (LND) quality or complication rates, remains inconclusive.

This study aimed to address these gaps by conducting a comprehensive comparison between RACS and LCS for CRC surgeries, focusing on key clinical and surgical parameters, including operative duration, blood loss, perioperative outcomes, and complication rates. In addition, our research’s innovation lay in its detailed analysis of specific surgical procedures, such as proctectomy and sigmoid colon resection, through hierarchical analysis, offering a more nuanced understanding of where RACS may offer advantages or face limitations. By critically evaluating these parameters, this research contributed to optimizing surgical decision-making in the management of CRC and helping to guide future clinical practice.

## Patients and methods

### Patient inclusion

Data on patients who underwent RACS and LCS from January 2021 to June 2024 at Tianjin Medical University Cancer Institute & Hospital (TJMUCH) were collected. Although the third-generation da Vinci robotic system was introduced at our center in 2019 to initiate robotic colorectal cancer surgeries, only cases from 2021 onwards were included to avoid data bias associated with the initial learning curve. The patients’ data were categorized into RACS and LCS groups. Patient inclusion criteria: (1) Preoperative diagnosis: CRC was confirmed via preoperative endoscopic pathology; (2) Operability of tumor: Patients with unresectable colon or high rectal tumors, or those with mid-to-low rectal cancer classified as T3 or higher or with regional lymph node metastasis, were required to undergo neoadjuvant therapy to enable complete tumor resection prior to surgery; (3) Surgical indications: The patient met the standard indications for surgery, with a single primary colorectal tumor or a single primary tumor accompanied by a resectable metastatic lesion. Additionally, the patient was able to tolerate RACS or LCS and had no severe complications that would contraindicate minimally invasive treatment; (4) Patient consent and willingness: Patients expressed a willingness to undergo either RACS or LCS, and provided informed consent for their participation in the procedure.

### Surgical approaches

RACS, using the da Vinci Si and Xi systems (Intuitive Surgical, USA), provides 3D high-definition imaging and multi-joint instruments, enabling precise operations by a two-person team (surgeon and first assistant). LCS, utilizing 2D imaging and straight instruments from KARL STORZ (Germany) and Stryker (USA), requires a three-person team (surgeon, first assistant, and second assistant). All patients undergoing RACS or LCS for colon cancer underwent a complete mesocolic excision (CME), while those with rectal cancer received a total mesorectal excision (TME). For patients with mid-to-low rectal cancer eligible for rectal-colonic anastomosis, the decision to perform an ileostomy was based on intraoperative assessment of anastomotic integrity, bowel perfusion, postoperative tension on the anastomotic site, and whether the patient had undergone neoadjuvant therapy. In both surgical methods, port placement and patient positioning were optimized to provide the surgeon with clear access to the surgical field, following the principles of CME and TME. In cases where significant intraoperative blood loss occurred, advanced local tumor staging was identified, or the patient exhibited abnormal vital signs during the minimally invasive procedure, the surgery was converted to open surgery.

### Data analysis

Key clinical and pathological characteristics, as well as perioperative outcomes, were compared between the two groups. Patient demographic data, including age and gender, as well as clinicopathological variables such as histological type, tumor differentiation, the American Joint Committee on Cancer (AJCC) pathological T stage (pT stage) and pathological N stage (pN stage), cancer nodule, and so on, were collected and analyzed. Surgical parameters, including lymph nodes dissection (LND), the duration of surgery, intraoperative blood loss, surgical site distribution (rectum and sigmoid colon), etc., were also assessed.

### Statistics

Data were analyzed with GraphPad Prism 10.0.2. Categorical variables were compared using the Chi-square test or Fisher’s exact test, while continuous variables were assessed using either the t-test or the Mann–Whitney U−test. Significance was determined with p-value less than 0.05.

## Results

### Basic characteristics between patients with RACS and LCS

A total of 225 patients with CRC were included in the study, including 82 cases of RACS and 143 cases of LCS. As shown in **Table 1**, there were statistically significant differences between RACS group and LCS group in terms of histological type (p = 0.015) and surgical site (p < 0.0001). Patients in RACS group had a higher prevalence of adenocarcinoma (40.24% vs. 27.97%) and a lower occurrence of mixed histological types (9.76% vs. 27.27%) than those in LCS group. In our medical center, the surgical sites of RACS primarily focused on the rectum and sigmoid colon, with rectal cancer accounting for 87.80% of the cases. However, there were no statistically significant differences between two groups in terms of age, gender, differentiation, pT stage, pN stage, presence of cancer nodules, and the administration of neoadjuvant chemotherapy (p > 0.05 for all).

**Table 1:** Basic clinicopathological data of patients undergoing robotic-assisted and laparoscopic colorectal surgeries.

Variables	RACS (82)	LCS (143)	*P value*
**Median age (years old)**	61	61	0.236
**Gender**			0.937
** Female**	36 (43.90%)	62 (43.36%)	
** Male**	46 (56.10%)	81 (56.64%)	
**Differentiation**			0.062
** Well**	1 (1.22%)	1 (0.70%)	
** Moderate**	68 (82.93%)	96 (67.13%)	
** Poor**	10 (12.20%)	33 (23.08%)	
** Other**	3 (3.66%)	13 (9.09%)	
**Histology**			**0.015**
** Adenocarcinoma**	33 (40.24%)	40 (27.97%)	
** Tubular adenocarcinoma**	39 (47.56%)	60 (41.96%)	
*Mixed adenocarcinoma	8 (9.76%)	39 (27.27%)	
** Other**	2 (2.44%)	4 (2.80%)	
**pT stage**			0.142
** T1**	5 (6.10%)	8 (5.59%)	
** T2**	21 (25.61%)	26 (18.18%)	
** T3**	47 (57.32%)	102 (71.33%)	
** T4**	5 (6.10%)	2 (1.40%)	
** Other**	4 (4.88%)	5 (3.50%)	
**pN stage**			0.788
** pN0**	55 (4.88%)	92 (64.3%)	
** pN1**	12 (14.63%)	28 (19.6%)	
** pN2**	11 (13.41%)	18 (12.6%)	
** Other**	4 (4.88%)	5 (3.50%)	
**Cancerous nodule**			0.964
** Negative**	69 (84.15%)	120 (83.92%)	
** Positive**	13 (15.85%)	23 (16.08%)	
**Neoadjuvant chemotherapy**			0.517
** Yes**	3 (3.66%)	8 (5.59%)	
** No**	79 (96.34%)	135 (94.41%)	
**Surgical location**			**< 0.0001**
** Rectum**	72 (87.80%)	79 (55.24%)	
** Sigmoid colon**	10 (12.20%)	29 (20.28%)	
** Other colon**	0 (0%)	35 (24.48%)	

RACS, robotic-assisted colorectal surgery; LCS, laparoscopic colorectal surgery; * Mixed adenocarcinoma including mucinous adenocarcinoma, signet ring cell carcinoma and other types of adenocarcinoma.

Bold p-values indicated statistically significant results (p < 0.05).

### Perioperative details, short-term outcomes, costs, and postoperative complications

The data in **Table 2** showed patients undergoing RACS had a significantly longer median surgery duration (218.5 minutes vs. 165 minutes, p < 0.0001) and greater median blood loss (100 mL vs. 50 mL, p < 0.0001) compared to those undergoing laparoscopic surgery (LCS). The median hospitalization cost was also higher in RACS, at 117,822 RMB (range: 78,225–1,627,735) compared to 78,174 RMB (range: 49,582–1,466,659) for LCS (p < 0.0001). Both groups had a similar median lymph node dissection count (RACS: 16, LCS: 15, p = 0.931) and median postoperative hospital stay (7 days, p = 0.475). Conversion to laparotomy, Clavien-Dindo classification, and postoperative complications, including anastomotic leakage, were low and comparable between groups (p > 0.05). The most common postoperative complications among patients with RACS or LCS are anastomotic leakage (3.66% for RACS; 4.20% for LCS) and bleeding (2.44% for RACS; 2.10% for LCS, **Table 3**).

**Table 2:** Intraoperative procedure details, immediate postoperative outcomes, and associated costs.

Variables	RACS (82)	LCS (143)	*P value*
**LND**			0.353
** < 12**	11 (13.41%)	26 (18.18%)	
** ≥ 12**	71 (86.59%)	117 (81.82%)	
**Median LND**	16 (5-35)	15 (3-56)	0.931
**Median duration of surgery (range, minutes)**	218.5 (135-460)	165 (80-420)	**< 0.0001**
**Median surgical blood loss (range, mL)**	100 (20-600)	50 (9-500)	**< 0.0001**
**Median postoperative hospitalization (range, days)**	7 (6-13)	7 (6-32)	0.475
**Conversion to laparotomy**			0.756
** Yes**	3 (3.66%)	5 (3.50%)	
** No**	79 (96.34%)	138 (96.50%)	
**Postoperative complications**			0.756
** Yes**	7 (8.54%)	14 (9.79%)	
** No**	75 (91.46%)	129 (90.21%)	
**Anastomotic leakage**			0.876
** Yes**	3 (3.66%)	6 (4.20%)	
** No**	79 (96.34%)	137 (95.80%)	
**Clavien-Dindo**			0.937
** 1**	75 (91.5%)	129 (90.2%)	
** 2**	4 (4.9%)	7 (4.9%)	
** 3**	3 (3.7%)	7 (4.9%)	
** 4**	0 (0%)	0 (0%)	
** 5**	0 (0%)	0 (0%)	
**Median total hospitalization cost (range, RMB)**	117,822 (78,225-16,2735)	78,174 (49,582-14,6659)	**< 0.0001**

RACS, robotic-assisted colorectal surgery; LCS, laparoscopic colorectal surgery; LND, lymph nodes dissection.

Bold p-values indicated statistically significant results (p < 0.05).

**Table 3:** Morbidity of postoperative complications.

Complications	RACS (7/82)	LCS (14/143)
**Anastomotic leakage**	3 (3.66%)	6 (4.20%)
**Abdominal infection**	1 (1.22%)	2 (1.40%)
**Bleeding**	2 (2.44%)	3 (2.10%)
**Urinary retention/infection**	1 (1.22%)	2 (1.40%)
**Intestinal obstruction**	0 (0%)	1 (0.70%)

RACS, robotic-assisted colorectal surgery; LCS, laparoscopic colorectal surgery.

### Clinicopathologic features of robotic vs. laparoscopic rectal and sigmoid resections

Since RACS in our medical center was only performed on the rectum and sigmoid colon, to prevent significant bias due to substantial differences in surgical sites, 109 patients who underwent LCS for the rectum and sigmoid colon were included for comparison with RACS. The operative time for patients undergoing robotic surgery was significantly longer than for those undergoing laparoscopic surgery (Median duration: 218.5 minutes for RACS vs. 160 minutes for LCS; p < 0.0001). The robotic-assisted surgery group had greater blood loss compared to the laparoscopic surgery group (Median blood loss: 100 mL for RACS vs. 50 mL for LCS; p < 0.0001). The RACS group had a higher proportion of rectal surgery patients (87.80% vs. 72.48%) and fewer sigmoid colon surgery patients (12.20% vs. 27.52%) compared to the LCS group (p = 0.01). The above two groups showed no statistically significant differences in age, gender, LND (above or below 12), number of LND, length of postoperative hospital stay, conversion to laparotomy, postoperative complications, anastomotic leakage, rectal surgical technique or ileostomy proportion (p > 0.05 for all, **Table 4**). In **Table 4**, among the 109 patients in the LCS group, 79 underwent rectal surgery, with 73 undergoing DIXON procedures, of which 33 had an ileostomy (45.2%, 33/73). In the RACS group, out of 82 patients, 72 underwent rectal surgery, with 67 undergoing DIXON procedures, of which 35 had an ileostomy (52.2%, 35/67).

**Table 4:** Clinicopathologic characteristics of patients performing robotic-assisted and laparoscopic surgery for rectal and sigmoid resection.

Variables	RACS (82)	LCS (109)	*P value*
**Median age (years old)**	61	61	0.210
**Gender**			0.985
** Female**	36 (43.90%)	48 (44.04%)	
** Male**	46 (56.10%)	61 (55.96%)	
**LND**			0.128
** < 12**	11 (13.41%)	24 (22.02%)	
** ≥ 12**	71 (86.59%)	85 (77.98%)	
**Median LND**	16 (5-35)	15 (3-42)	0.144
**Median duration of surgery (range, minutes)**	218.5 (135-460)	160 (80-420)	**< 0.0001**
**Median surgical blood loss (range, mL)**	100 (20-600)	50 (10-500)	**< 0.0001**
**Median postoperative hospitalization (range, days)**	7 (6-13)	7 (6-32)	0.806
**Conversion to laparotomy**			0.949
** Yes**	3 (3.66%)	3 (2.75%)	
** No**	79 (96.34%)	107 (97.25%)	
**Postoperative complications**			0.761
** Yes**	7 (8.54%)	8 (7.34%)	
** No**	75 (91.46%)	101 (92.66%)	
**Anastomotic leakage**			0.962
** Yes**	3 (3.66%)	5 (4.59%)	
** No**	79 (96.34%)	104 (95.41%)	
**Surgical location**			**0.01**
** Rectum**	72 (87.80%)	79 (72.48%)	
** Sigmoid colon**	10 (12.20%)	30 (27.52%)	
**Surgical technique**			0.878
** Miles**	5 (6.94%)	6 (7.59%)	
** Dixon**	67 (93.06%)	73 (92.41%)	
**Ileostomy**			0.076
** Yes**	35 (42.68%)	33 (30.28%)	
** No**	47 (57.32%)	76 (69.72%)	

RACS, robotic-assisted colorectal surgery; LCS, laparoscopic colorectal surgery; LND, lymph nodes dissection.

Bold p-values indicated statistically significant results (p < 0.05).

### Comparing our robotic-assisted surgery data with published results

As was shown in **Table 5**, the median operative time for RACS at TJMUCH was 218.5 minutes. This was slightly longer than the average time reported by Gansu Provincial Hospital in China (205.9 minutes) and exceeds the median time at most hospitals in China (180 minutes). However, it was shorter than the median operative time at Ljubljana medical center in Slovenia (262 minutes) or Illinois medical center in USA (347 minutes). The median intraoperative blood loss of patients undergoing RACS at TJMUCH (100mL) was lower than the average blood loss at Gansu Provincial Hospital in China (147.8mL), the median blood loss at hospitals in most regions of China (104mL), and the median blood loss at a hospital in the state of Illinois, USA (150mL). Moreover, RACS for rectal cancer accounted for a very high proportion in Chinese and international medical centers. The RACS proportion of rectal cancer cases at TJMUCH was generally the highest compared to other medical centers. The mean total hospitalization cost for robotic surgery at our center (117,584 RMB) was higher than that of Gansu Provincial Hospital (84,990 RMB).

**Table 5:** Comparison of RACS duration, intraoperative blood loss, surgical site and surgical cost at TJMUCH with other literature.

Variables	TJMUCH	Gansu Provincial Hospital, China (10)	Nationwide Multicenter, China (11)	Ljubljana Medical Center, Slovenia(12)	Illinois Medical Center, USA (13)	Multiple Countries (14)
**Number of patients**	82	271	10329	83	44	1635
**Median/mean duration (minutes)**	218.5	205.9	180	262	347	–
**Median/mean blood loss (mL)**	100	147.8	104	–	150	–
**Proportion of proctectomy**	72 (87.80%)	197 (72.69%)	4854 (46.99%)	–	–	1151 (70.40%)
**Mean surgical cost (RMB)**	117,584	84,990	–	–	–	–

TJMUCH, Tianjin Medical University Cancer Institute & Hospital; RACS, robotic-assisted colorectal surgery.

### Comparison of images between RACS and LCS for proctectomy

**Figure 1:**
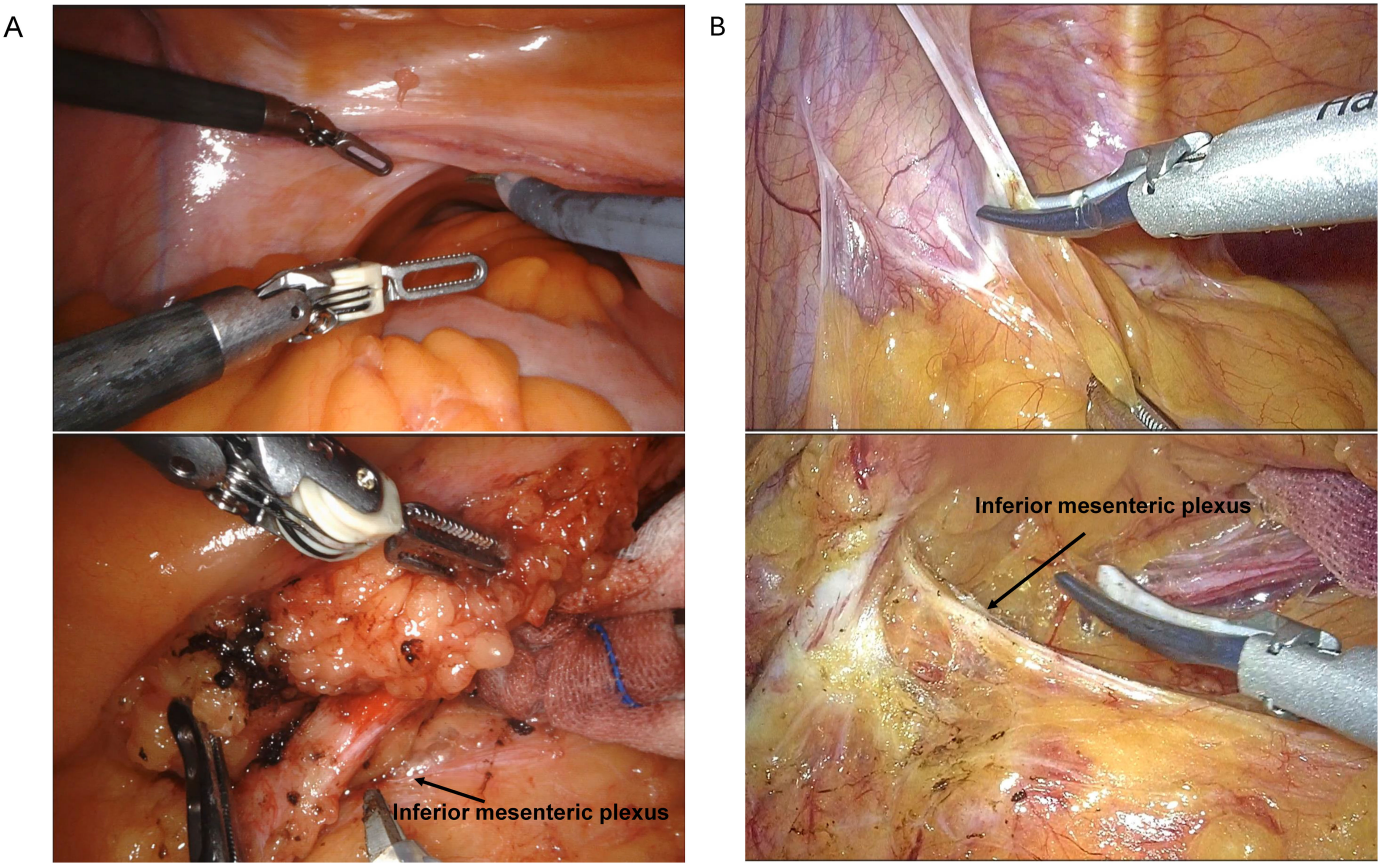
Comparison of surgical fields between RACS **(A)** and LCS **(B)**. RACS, robotic-assisted colorectal surgery; LCS, laparoscopic colorectal surgery.

During RACS, a 3D visual field is used, providing a clearer view compared to the 2D visual field of LCS. However, based on the images in **Figures 1A, B**, our center believes that LCS offers advantages in terms of the tissue retraction, the dissection field around the root of the inferior mesenteric artery, and the tension on the inferior mesenteric nerves compared to RACS.

## Discussion

In this study, we conducted a comprehensive comparison between RACS and LCS in a cohort of 225 patients with CRC. Our findings reveal several key differences and similarities between these two surgical modalities. Firstly, our analysis demonstrated statistically significant differences between the RACS and LCS groups in terms of duration of surgery, intraoperative blood loss, surgical costs, and surgical site distribution. The operative time was notably longer in the RACS group compared to the LCS group. This finding aligns with existing literature, which consistently reports prolonged operative times for robotic surgery (15–17). Despite this, the extended operative time does not appear to translate into increased postoperative morbidity, as the rates of complications such as anastomotic leakage and bleeding were comparable between the two groups. A meta-analysis of RACS versus LCS by YANG et al. did not confirm a significant difference in operative time between the two groups (18). The primary reason for these controversial findings may be the setup and docking of the robotic system and the learning curve associated with using the robotic system.

Additionally, the median hospitalization cost was significantly higher for RACS than for LCS, a result consistent with findings from previous studies (18). This cost difference likely reflects the higher setup and operational expenses associated with robotic technology, which can impact overall healthcare expenditures.

Interestingly, the RACS group also experienced greater intraoperative blood loss than the LCS group. Hu et al. also reported that more blood loss volume was found among patients performing RACS compared to those undergoing LCS (10). Farah et al. summarized the data of colectomy-targeted American College of Surgeons-National Surgical Quality Improvement Program (ACS-NSQIP) database (2015–2020) and found patients with RACS had higher bleeding transfusion occurrence for low anterior resection dataset than those with LCS, but there was no significant difference in bleeding transfusion occurrence for right or left colectomy dataset between RACS and LCS (19). However, it is worth noting that the overall blood loss in both groups remained within acceptable clinical ranges, and the difference did not appear to significantly impact patient outcomes, such as postoperative recovery time or complication rates.

Our analysis also highlighted the distinct focus on rectal and sigmoid colon surgeries within our medical center’s RACS program, with rectal cancer comprising the majority of cases (11, 12, 18). This distribution may reflect the perceived advantages of robotic systems in performing intricate pelvic surgeries where enhanced 3D visualization and instrument dexterity are crucial (15, 20, 21). However, our findings indicate that while robotic systems offer superior visual clarity, LCS may offer certain benefits in tissue retraction and exposure of the dissection field, especially around the inferior mesenteric artery and for nerve tension management in proctectomy. This suggests that while robotic systems offer superior visual clarity, the effectiveness of tissue manipulation and anatomical dissection in LCS cannot be overlooked.

When comparing our RACS data with those from other institutions, we observed that the median operative time at our center was slightly longer than that reported by other centers in China but shorter than that in some international centers, such as Slovenia (10–13). The intraoperative blood loss at our center was also lower than the reported averages from other hospitals in China and the USA, highlighting the efficiency of our surgical team in minimizing blood loss during RACS (11, 13).

This study is subject to certain limitations. Firstly, this was a retrospective study, which led to some data bias. A prospective study design would help to reduce this selection bias. Secondly, in future studies, we plan to increase the sample size and enhance the stability of the evidence by adopting a multicenter approach. This approach will help to strengthen the validity and generalizability of the findings. Thirdly, while our results are aligned with previous literature, some limitations persist in the breadth and scope of our data compared to larger, multicenter datasets. In addition, another limitation of this study is the lack of long-term clinical outcomes. In future research, we aim to include the collection and statistical analysis of such outcomes to provide a more comprehensive evaluation.

Overall, our findings suggest that while RACS offers certain advantages, particularly in visual clarity and dexterity in confined surgical spaces, LCS remains a strong contender, especially in terms of tissue retraction and field exposure for proctectomy. The decision between RACS and LCS should be guided by the specific clinical context, surgeon expertise, and patient characteristics. Further multicenter studies with larger sample sizes and long-term follow-up are needed to validate these findings and refine the selection criteria for RACS versus LCS.

## Data Availability

The raw data supporting the conclusions of this article will be made available by the authors, without undue reservation.
